# On Some Minor Anal and Rectal Ailments.—I

**Published:** 1895-02-23

**Authors:** W. McAdam Eccles

**Affiliations:** Assistant Surgeon West London Hospital and City of London Truss Society, Surgeon St. Marylebone General Dispensary, and Senior Assistant Demonstrator of Anatomy St. Bartholomew's Hospital


### MEDICAL PROGRESS AND HOSPITAL CLINICS.

[*The Editor will be glad to receive offers of co-operation and contributions from members of the profession. All letter should be addressed to* THE EDITOR, THE LODGE, PORCHESTER SQUARE, LONDON, W.]

### ON SOME MINOR ANAL AND RECTAL AILMENTS.—I.

By W. McADAM ECCLES, M.B., M.S. (Lond.), F.R.C.S. (Eng.), Assistant Surgeon West London Hospital and City of London Truss Society, Surgeon St. Marylebone General Dispensary, and Senior Assistant Demonstrator of Anatomy St. Bartholomew's Hospital.

IT is a fact which has been the experience of all practitioners that there are several pathological conditions of the anus and rectum which occasion an amount of annoyance altogether out of proportion to the lesion.

Among these may be enumerated the following: Anal fissure and anal ulcer, superficial anal abscess, pruritus ani, anal condylomata, vascular patches of the rectal mucous membrane, and superficial ulceration of the rectal mucous membrane. Each of these deserves close attention, for many patients endure much suffering from one or other of them.

A thorough examination of the region is always absolutely necessary; and although at times it is difficult to obtain without firmness and tact on the part of the surgeon, the gratification caused by the succeeding successful, because intelligent, treatment is pleasing both to the sufferer and his medical attendant. I have not infrequently been asked to treat cases where patients have had some such anal trouble for a considerable time, but which has been overlooked on account of the objection, often natural, to visual inspection. In certain subjects it is advisable to have an anæsthetic administered during the exploration.

Let us briefly review the above minor but highly interesting disorders.

**Anal Fissure and Anal Ulcer** are closely associated, and they are by no means infrequent troubles. It is only natural that slight tears and excoriations about the anus must very often occur, when we think of the anatomy of the part, and the habitual stretching which the anal margin has to undergo in defæcation, especially when the motions are much constipated.

The conditions are typically present in anæmic women, who so commonly have infrequent evacuation of the bowels. Now and again one of these little fissures, which generally rapidly heal, becomes irritated and refuses to disappear, and consequently a small anal ulcer is formed. This is is extremely liable to be accompanied by a distinctive train of symptoms. The patient is worried, in fact often very severely, during or just after defæcation by a marked sharp hot smarting or cutting pain about the anus, and radiating to the parts around. This will usually last for some time, and is then frequently replaced by a dull, heavy, aching pain, which in some cases may continue until the bowels are next opened. It will be noticed that a vicious circle is thereby set up—constipation, itself by no means rarely the outcome of the want of a proper regular daily attempt to evacuate the contents of the rectum, produces the small lesion of an anal ulcer, and this, on account of the pain it gives rise to, causes the sufferer to postpone defæcation for an inordinate length of time, so rendering a continuance of the original mischief all the more likely.

It is worth while here remarking that an anal ulcer may be an adequate cause of dyspareunia in women. There is no doubt that all this pain which is associated with such ulcers is chiefly due to exposure of the highly sensitive nerve-endings of the part, and to reflex spasm of the external sphincter-ani muscle. Patients may go on with this torture, and with the accompanying slight loss of blood and discharge, for an astonishing length of time, until an examination by the surgeon reveals, when the folds at the margin of the anus are separated, a typical ulcer but frequently of quite a small extent.

**Treatment.**—After discovery of the cause, treatment is usually a comparatively simple matter. If the ulcer be of recent origin and quite superficial it will not uncommonly heal if a soft daily motion be secured. Whatever has been found to suit the patient in the way of a laxative may be ordered, but a third to a half a tumblerful of Friedrichshalle water diluted with an equal quantity of hot water and taken early in the morning on an empty stomach, answers admirably.

All irritation in cleansing the parts after an evacuation must be carefully avoided. Some wet absorbent wool or soft sponge is the best material to employ for this purpose. Some recommend in addition a simple ointment as a topical application.

If recovery does not quickly, say in a fortnight or three weeks, follow this course of treatment, or if the affection is of some standing, or the ulcer deep, exposing some muscular fibres of the sphincter, then an operation of the simplest character will effectually put an end to the trouble.

The bowels having been freely purged beforehand, the patient is anæsthetised, and the sphincter thoroughly dilated. After this the floor of the ulcer is to be incised, the sphincter muscle at the same time being cut through for about a third of its thickness. The method practically in all cases brings speedy relief, the bowels being opened two days after with scarcely any pain. The patient need not be confined to bed for more than three or four days, but should take care to avoid any return of the causes which are likely to reproduce his trouble.

**Superficial Anal Abscess.**—The glands around the margin of the anus have a great tendency to become inflamed, and suppuration not infrequently occurs. These small abscesses may lead to annoying consequences unless actively treated. They may be the starting point of ulceration, or may cause a small fistula which refuses to heal. As soon as ever one is discovered the patient should be told that a small incision is imperative, and it may be rendered perfectly painless by the use of the ethyl chloride spray. This early incision is of the utmost importance, and usually leads to a rapid return of the parts to their normal condition.

The remaining affections will be dealt with in another paper.

